# Schizophrenia and Heart Health: Are Antipsychotics a Friend or Foe?

**DOI:** 10.3390/jpm14080814

**Published:** 2024-07-31

**Authors:** Minodora Andor, Liana Dehelean, Diana Aurora Arnăutu, Marioara Nicula Neagu, Daciana Nistor, Minodora Marinela Manea, Ana-Maria Romosan, Nilima Rajpal Kundnani

**Affiliations:** 1 Medical Semiology II, Internal Medicine I Department, “Victor Babeş” University of Medicine and Pharmacy, 2 E. Murgu Square, 300041 Timişoara, Romania; 2 Multidisciplinary Heart Research Centre, “Victor Babeş” University of Medicine and Pharmacy, 2 E. Murgu Square, 300041 Timişoara, Romania; 3 Psychiatry, Neurosciences Department, “Victor Babeş” University of Medicine and Pharmacy, 2 E. Murgu Square, 300041 Timişoara, Romania; 4 Discipline of Physiology, Faculty of Bioengineering of Animal Resources, University of Life Sciences “King Mihai I”, 300041 Timișoara, Romania; 5 Department of Functional Sciences, Physiology, Center of Immuno-Physiology and Biotechnologies (CIFBIOTEH), “Victor Babes” University of Medicine and Pharmacy, 300041 Timisoara, Romania; 6 Centre for Gene and Cellular Therapies in Cancer, 3000723 Timisoara, Romania; 7 Psychology, Medical Education Department, “Iuliu Haţieganu” University of Medicine and Pharmacy, V. Babeş Street, 400012 Cluj-Napoca, Romania; 8 Discipline of Internal Medicine and Ambulatory Care, Prevention and Cardiovascular Recovery, Department VI—Cardiology, “Victor Babes” University of Medicine and Pharmacy, 300041 Timisoara, Romania; knilima@umft.ro; 9 Research Centre of Timisoara Institute of Cardiovascular Diseases, “Victor Babes” University of Medicine and Pharmacy, 300041 Timisoara, Romania

**Keywords:** Brief Psychiatric Rating Scale Extended (BRSE), long-acting injectable (LAI) antipsychotics, cardiovascular risk factors, schizophrenia

## Abstract

Schizophrenia is one of the most disabling of the psychiatric diseases. The Brief Psychiatric Rating Scale Extended (BRSE) is used to evaluate the severity of psychiatric symptoms. Long-acting injectable (LAI) antipsychotics are commonly used and are preferred over oral antipsychotic medications. A two-center-based cross-sectional study was performed on 130 patients diagnosed with schizophrenia or schizoaffective disorder based on the International Classification of Diseases 10 criteria. We studied the relation between the development of cardiovascular risk factors and the antipsychotic medication that was administered in these patients. Our study demonstrates strong links between several cardiovascular risk factors and the duration of psychosis; the duration of the LAI antipsychotic treatment; the duration between the onset of the disease and the start of LAI antipsychotic treatment; and the use of specific LAI antipsychotic medications.

## 1. Introduction

Schizophrenia is a serious psychiatric disorder where the ability to exhibit clear thinking is affected, along with the tendency to act or behave normally or to feel. It is one of the most common mental illnesses found in day-to-day practice [1,2]. The early development of the brain is affected due to heterogeneous genetic abnormalities and neurobiological changes [3]. The main key symptoms include hallucinations, disordered thoughts, delusions, a lack of motivation to accomplish goals, disturbances in sleep patterns, slow movements, motor and cognitive impairments, poor grooming, poor hygiene, changes in body language and emotions, and difficulties in maintaining social relationships. Although the symptoms differ from case to case, they can be grouped into three broad categories: positive (psychotic), negative (deficits), and cognitive [2]. Usually, patients suffering from this mental disorder are diagnosed in adolescence or early adulthood. Monitoring developmental milestones can help to identify children suffering from this illness at a much younger age. Moreover, inappropriate or unusual behaviors, together with cognitive impairment, are present in childhood but are often missed by the family and pediatricians or family doctors [3,4]. The presence of multiple symptoms indicates the advancement of the disease to a severe stage. Early life stress or parental factors that affect the environment of the child may lead to disruptions in the development of the brain, which, if left untreated, may lead to severe forms of schizophrenia.

The Brief Psychiatric Rating Scale Extended (BRSE) is frequently used in psychiatric cases to evaluate the severity of psychiatric symptoms. The scale is based on a doctor-to-patient interview and includes an evaluation of different behavioral patterns observed during the past 2–3 days. The patient’s family observation reports can also be considered. It is a points-based questionnaire, with scores ranging from 0 to 7 points for every question. A score of “0” is negative or indicates the absence of symptoms and “7” denotes the presence of extremely severe symptoms [5].

In a meta-analysis conducted by K. Hagi et al. [6], the existence of an association between schizophrenia patients having metabolic syndrome, hypertension, or diabetes and cognitive impairment was described. Cardiovascular risk factors play an important role, leading to cognitive decline in psychosis cases. Premature mortality is commonly witnessed in psychiatric patients, with the predominant culprit being the cardiovascular system.

Medications used to treat schizophrenia can help to control the psychotic symptoms but are not very effective in improving the occupational, cognitive, or social functioning of the patient. Cognitive behavioral therapy (CBT), cognitive remediation (CR), and support systems for education and employment are some of the psychosocial interventions that can help to improve the overall outcomes. Since there is a significant delay in diagnosis, in order to treat patients efficiently for risk mitigation, a global strategic approach is required, involving a psychiatrist and psychotherapist, along with the support of the family and the family physician.

Long-acting injectable (LAI) antipsychotics are preferred nowadays to overcome patient treatment adherence issues, which are witnessed with oral antipsychotic medications. LAI antipsychotics ensure a sustained effect upon target neuroreceptors that transmit specific neurochemicals, and this forecloses the need for oral medications on a regular basis, an aspect that patients diagnosed with schizophrenia and related conditions often find difficult. The benefit is that they allow the slow release of the drug into the bloodstream, which helps in reducing the frequency of administration. Risperidone, olanzapine, aripiprazole, and paliperidone are all frequently used LAI antipsychotics that are approved by the FDA and are named second-generation LAI antipsychotics. Nine different forms are available of these four drugs. These injections are costly but worthwhile as not only do they help in treatment adherence but they also improve patient outcomes. The route of administration is intramuscular and, differing with the drug preparation, gluteal or deltoid, with the latter providing better absorption. Similarly, the effectiveness of each shot can vary from weeks to months depending on the different preparations [7].

The commencement of LAIs in patients may be delayed due to certain reasons, such as a lack of information regarding the drug’s pharmacokinetics and dose selection [8], an overestimation of patient adherence to OA [9], and a lack of knowledge and updates regarding the benefits of second-generation LAI antipsychotics versus first-generation drugs, as well as oral antipsychotics [10,11]. Antipsychotics are lipophilic drugs and hence some drug is stored in the body’s lipid deposits, which may accumulate over time [12]. This is of concern since any adverse effect that appears after LAI administration will likely persist over a longer duration. As a discouraging aspect, there is evidence of injection-related adverse events and overall negative perceptions of the safety of LAI versus oral antipsychotics (OA) due to the slightly higher occurrence of extrapyramidal symptoms and tardive dyskinesia in LAI [13,14]. However, the perceived safety of OA use is also countered by their poor adherence and underdosing [15].

Mental illness in the form of schizophrenia and its range of symptoms has an adverse impact on patients’ cognition, behavior, and emotions, as well as on the performance of daily life activities [16]. There is a fivefold higher risk of cardiovascular mortality and sudden cardiac death (SCD) in patients with SMI [17]. This holds true across both sexes, all ages, and all ethnic groups [18]. Surprisingly, the death rates related to cardiovascular conditions in patients suffering from schizophrenia and other mental illnesses have been increasing [19]. This calls for attention to and a focus on analyzing the link between schizophrenia’s evolution, treatment, and cardiovascular risks.

## 2. Material and Methods

We performed a two-center-based (Timisoara and Cluj, Romania) cross-sectional study on 130 patients that presented to our ambulatory services and were diagnosed with schizophrenia or schizoaffective disorders. Patients were diagnosed based on the International Classification of Diseases 10 criteria. Patients with a recent acute psychotic episode in the last 2 months (decompensated), patients with oral antipsychotic medication (compliance with medication may be questionable), and patients with a previously diagnosed cardiovascular disease were excluded. Patients who were placed on more than one LAI and those who were given multiple LAIs over their medication history were also excluded from this study.

The following parameters were evaluated for the entire group of patients: demographic data, clinical data (age of onset of psychosis, duration of psychosis, duration of injectable antipsychotic treatment, and duration of pre-LAI treatment), and the severity of the psychiatric symptoms using the Brief Psychiatric Rating Scale Extended (BRSE), as well as data after clinical examination and paraclinical findings (blood tests, electrocardiogram, echocardiography, and speckle tracking analysis).

The IBM Statistics for Windows program, version 20, was used to analyze the data. As the Shapiro–Wilk test for normality of distribution showed a non-Gaussian distribution of the data, differences between groups were checked using non-parametrical tests (the Kruskal–Wallis test). Potential associations between the symptoms, the duration of the disease, and the cardiac EF were assessed using Spearman’s correlation coefficients. For all statistical tests, the level of significance was considered 0.05 and all results were two-tailed.

The study was approved by the Scientific Research Ethics Committee of “Victor Babes” University of Medicine and Pharmacy, Timisoara, Romania (approval number 19/2015) and was conducted in accordance with the Helsinki Declaration. Written informed consent was obtained as a part of the routine procedure from all patients/relatives/legal caretakers undergoing treatment at our university hospitals for further research and educational purposes.

## 3. Results

Cardiovascular risk factors were studied in patients with schizophrenia or schizoaffective disorder, depending on the type of medication that was administered.

The entire group of patients was divided into four subgroups, depending on the LAI antipsychotic medication administered to them, as shown in Table 1.

Table 2 shows the number of patients in each dosage category for each antipsychotic drug. It is evident that most patients were prescribed olanzapine (N = 38), with the most dose variations (four), and there was a single dosage pattern with regard to prescribing aripiprazole, i.e., 400 mg/month.

Table 3 shows the number of months that passed since the onset of psychosis, the months since LAI treatment initiation, and the gap between the onset of psychosis and the initiation of LAI among the different age groups.

Table 4 shows the number of patients in each age group that were given LAI antipsychotics, with most patients receiving olanzapine.

When using the Kruskal–Wallis test, followed by the Dunn–Bonferroni post hoc test, we found no significant differences between the four groups regarding the

- Age (*p* = 0.27);
- Age at the onset of the disease (*p* = 0.11);
- Total duration of the disease (*p* = 0.38);
- Duration of pre-LAI antipsychotic treatment (*p* = 0.52).

**Figure 1:**
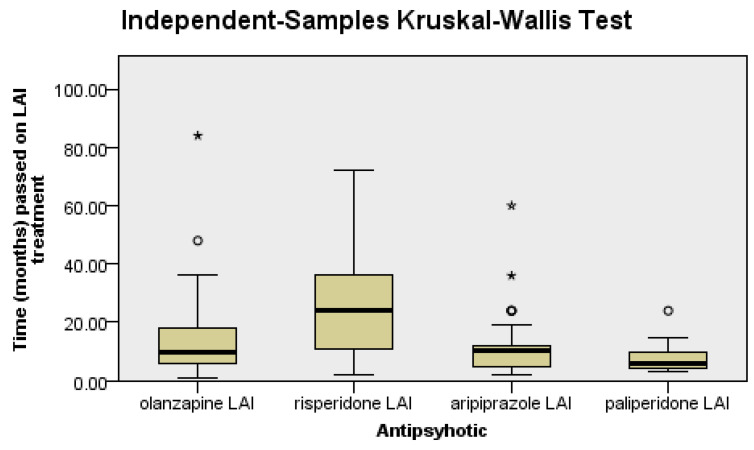
Risperidone patients have a longer duration of treatment than the rest of the patients.

There were significant differences between the groups regarding the LAI antipsychotic treatment duration (*p* < 0.0001), as follows: patients on risperidone had a significantly longer LAI antipsychotic treatment duration than patients on olanzapine (*p* = 0.04), aripiprazole (*p* = 0.03), or paliperidone (*p* < 0.0001), since it was the first LAI second-generation antipsychotic introduced in clinical practice (Figure 1).

Data regarding various cardiovascular risk factors are shown in Table 5.

Cardiovascular risk factors depending on the medications consumed are represented in Table 6. No significant differences were found between the four groups regarding the presence of abdominal obesity (χ^2^ = 2.39, *p* = 0.49).

Similarly, no significant differences were found between the four groups regarding the presence of hypertension (χ^2^ = 2.67, *p* = 0.44).

Significant differences were also not found between the four groups regarding the presence of hypertriglyceridemia (χ^2^ = 3.82, *p* = 0.28).

There were statistically significant differences witnessed between the groups in terms of hyperglycemia (χ^2^ = 13.43, *p* = 0.004): patients on olanzapine and risperidone had significantly more frequent hyperglycemia compared to those on aripiprazole or paliperidone.

There were statistically significant differences noted between all four groups regarding hypo-HDL (χ^2^ = 17.80, *p* < 0.0001): patients on olanzapine and risperidone had significantly more frequent hypo-HDL compared to those on aripiprazole or paliperidone.

There were no significant differences between the four groups regarding smoking habits (χ^2^ = 1.45, *p* = 0.69).

Regarding echocardiographic changes, we found the following.

There were statistically significant differences between the four groups regarding the presence of hypokinetic disorders (χ^2^ = 8.98, *p* = 0.03): patients on risperidone presented significantly more frequent hypokinetic disorders compared to those on olanzapine, aripiprazole, or paliperidone; see Table 7.

There were no significant differences between the four groups regarding the presence of valvular dysfunction (χ^2^ = 12.82, *p* = 0.61) and also no significant differences between the four groups regarding the presence of pulmonary hypertension (χ^2^ = 5.45, *p* = 0.14), but there were statistically significant differences between the four groups regarding the presence of diastolic dysfunction (χ^2^ = 8.77, *p* = 0.03): patients on risperidone and aripiprazole presented significantly more frequent diastolic dysfunction compared to those on olanzapine or paliperidone.

Regarding the duration of the psychosis, the duration of the antipsychotic treatment, and the duration between the onset of the disease and the start of the antipsychotic treatment, the following were found.

**Figure 2:**
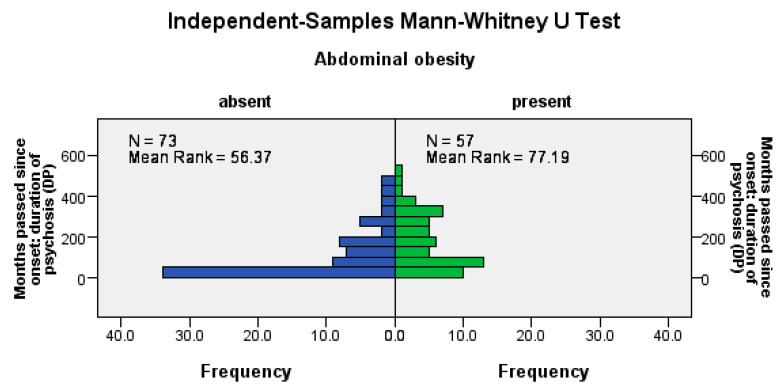
Abdominal obesity correlates positively with the duration of the disease, the time before the start of the antipsychotic treatment, and the duration of the antipsychotic treatment.

Abdominal obesity was significantly more frequent among patients with a longer psychosis duration (U = 1414, Z = −3.13, *p* = 0.002), among patients with a longer LAI antipsychotic duration (U = 1515, Z = −2.66, *p*= 0.008), and among those with a longer pre-LAI antipsychotic duration (U = 1474, Z = −2.85, *p* = 0.004); see Figure 2.

Hyperglycemia was significantly more frequent among patients with a longer LAI antipsychotic duration (U = 1267, Z = −3.25, *p* = 0.001), with no significant differences between patients with a longer DP and those with a shorter DP regarding hyperglycemia (*p* = 0.08) and with no significant differences between patients with a longer pre-LAI antipsychotic duration and those with a shorter pre-LAI antipsychotic duration regarding hyperglycemia (*p* = 0.16).

HTN was significantly more frequent among patients with a longer pre-LAI antipsychotic duration (U = 2326, Z = −2.35, *p* = 0.04). There were significant differences between patients with a longer DP and those with a shorter DP regarding HTN (*p* = 0.55) but no significant differences between patients with a longer LAI antipsychotic duration and those with a shorter LAI antipsychotic duration regarding HTN (*p* = 0.45).

No significant differences were found between patients with a longer DP, pre-LAI antipsychotic duration, and LAI antipsychotic duration and those with a shorter DP, pre-LAI antipsychotic duration, and LAI antipsychotic duration regarding the frequency of hypertriglyceridemia (*p* > 0.05), regarding the frequency of hypo-HDL cholesterol (*p* > 0.05), and in terms of the smoking frequency (*p* > 0.05).

Concerning the correlation between the duration of psychosis, the time before the start of the antipsychotic treatment, and the duration of LAI antipsychotic treatment and echocardiographic changes, we can state the following.

**Figure 3:**
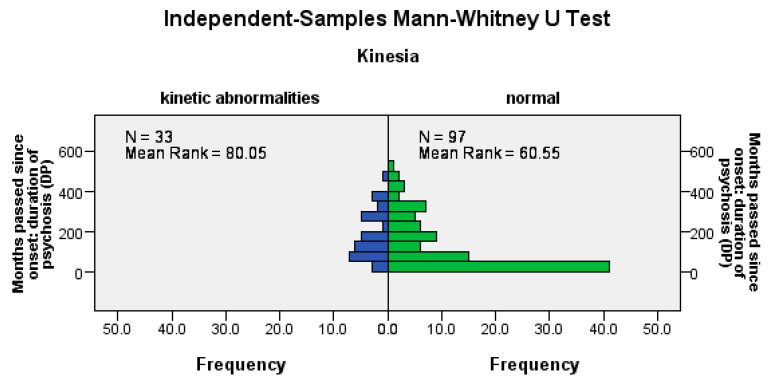
The occurrence of hypokinetic disorders correlates positively with the duration of the disease, the time before the start of the LAI antipsychotic treatment, and the duration of the treatment.

Hypokinesia disorders were significantly more frequent among patients with a longer DP (U = 1120.5, Z = −2.57, *p* = 0.01), among patients with a longer LAI antipsychotic duration (U = 1162.5, Z = −2.66, *p*= 0.02), and among those with a longer pre-LAI antipsychotic duration (U = 1178, Z = −2.26, *p* = 0.02); see Figure 3.

Valvular changes were significantly more frequent among patients with a longer pre-LAI antipsychotic duration (U = 1352, Z = −3.16, *p* = 0.002). Valvular changes were significantly more frequent among patients with a longer DP (U = 1375, Z = −3.05, *p* = 0.002). No significant differences were found between patients with a longer LAI antipsychotic duration and those with a shorter LAI antipsychotic duration regarding the presence of valvular changes (*p* = 0.52).

**Figure 4:**
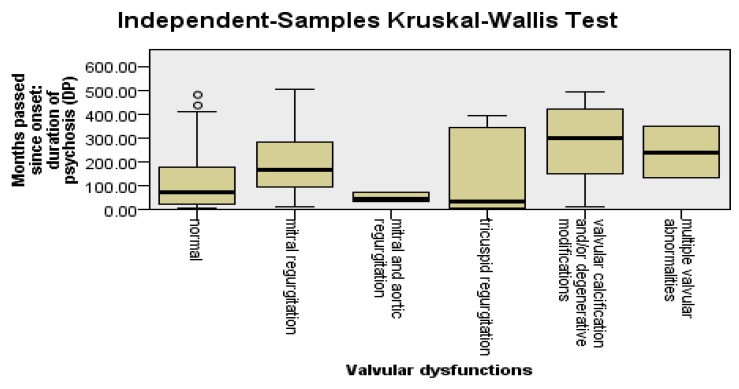
Valvular changes are more frequent among patients with DP and a longer pre-LAI antipsychotic duration.

To detect the types of valvular changes that were more frequent among patients with DP and a longer pre-LAI antipsychotic duration, we applied the Kruskal–Wallis test, followed by the Dunn–Bonferroni post hoc test. Mitral regurgitation was found to be significantly more frequent among patients with DP (*p* = 0.03) and the pre-LAI antipsychotic duration (*p* = 0.03) was longer. The other types of valvular changes did not have statistically significant relevance in relation to DP, the pre-LAI antipsychotic duration, or the LAI antipsychotic duration; see Figure 4.

Diastolic dysfunction was significantly more frequent among patients with a longer pre-LAI antipsychotic duration (U = 925, Z = −4.68, *p* < 0.0001). Diastolic dysfunction was significantly more frequent among patients with a longer DP (U = 911.5, Z = −4.75, *p* < 0.0001). There were no significant differences between patients with a longer LAI antipsychotic duration and those with a shorter LAI antipsychotic duration regarding diastolic dysfunction (*p* = 0.62).

No significant differences were found between patients with DP, a pre-LAI antipsychotic duration, and a longer LAI antipsychotic duration and those with DP, a pre-LAI antipsychotic duration, and a shorter LAI antipsychotic duration regarding the frequency of HTP (*p* > 0.05).

## 4. Discussion

Sudden cardiovascular death is up to five times more common in patients with schizophrenia and other mental illnesses than in the general population [20]. More than 60% of patients with schizophrenia and other mental illnesses have had undetected cardiovascular disease before any grave cardiovascular event [21], and this is significantly higher than in the general population. The higher mortality rate [17,18] and its increasing trend [19] have raised concerns regarding a better understanding of the link between the management of schizophrenia and cardiovascular risk factors. There is a clearly higher mortality rate in patients with schizophrenia and other mental illnesses, even without a high coronary artery calcification score [22,23], which may usually have added predictive value. Several studies have presented work in this direction. Our study is one of the first in Romania to establish a relationship between the treatment of schizophrenia and the cardiovascular risk.

Our study demonstrated a strong relationship between several cardiovascular risk factors and the duration of psychosis; the duration of the LAI antipsychotic treatment; the duration between the onset of the disease and the start of LAI antipsychotic treatment; and the use of specific LAI antipsychotic medications. These are discussed in the subsequent paragraphs.

Abdominal obesity was more frequent among patients with a longer duration of psychosis (DP). Hyperglycemia was more frequent among patients with a longer duration before the onset of LAI antipsychotic use. Patients on olanzapine and risperidone had significantly more frequent hyperglycemia compared to those on aripiprazole or paliperidone. Patients on olanzapine and risperidone had more frequent low HDL levels that posed a cardiovascular risk. Social deprivation is linked to the poor control of cholesterol, poor management of blood glucose levels, and poor outcomes in patients with coronary artery disease [24,25]. It is to be noted here that patients having higher BRSE scores with or without treatment resistance would require higher dosages of LAIs [26].

Hypertension was more frequent among patients with a longer duration between the onset of the disease and the start of the LAI antipsychotic treatment. According to old and new literature [27,28], there is a very high rate of unrecognized myocardial infarction in patients with schizophrenia and psychosis. Factors contributing to myocardial infarction in such patients include hyperglycemia, low HDL levels, and hypertension, but are not limited to these alone. A change in pain threshold with decreased pain sensitivity is one mechanism to which the high cardiovascular risk may be attributed in schizophrenia patients [29]. This may explain the late diagnosis of cardiovascular comorbidities in such patients.

Antipsychotic drugs are linked with arrhythmogenic risks in terms of QTc interval prolongation, ventricular arrhythmias, polymorphic ventricular tachycardia torsades de pointes, and sudden cardiac arrest [30]. Antipsychotic drugs are also linked with a reduced left ventricular ejection fraction in patients with schizophrenia [31]. In our study, the occurrence of hypokinetic heart disorders correlated positively with the duration of psychosis, the duration of the LAI treatment, and the duration between the onset of the disease and the start of LAI treatment. In our study, patients on risperidone presented more frequent hypokinetic heart disorders. Echocardiographic findings of valvular changes as well as of diastolic dysfunction were more frequent among patients with a longer duration of psychosis and a longer pre-LAI duration. Another study conducted by Dahelean et al. in 2018 [32] also observed similar findings. Patients on risperidone had significantly more frequent regional myocardial contractility abnormalities and left ventricular diastolic dysfunction than those on olanzapine. Hypokinesia, valvular findings, and diastolic dysfunction can easily lead to ventricular arrhythmias. The pro-arrhythmic effects of psychotropic drugs including antipsychotics and antidepressants can contribute to the increased arrhythmogenicity in patients with schizophrenia [33]. The development of myocardial impairment in schizophrenia patients on long-term treatment may be due to the high prevalence of cardiovascular risk factors and may be generated by the disease itself [34]. Moreover, a delay in initiating the LAI treatment of schizophrenia patients is a statistically relevant risk factor for LV diastolic dysfunction and overall cardiovascular impairment [34]. Our study also demonstrated that LV diastolic dysfunction was significantly more frequent among patients with a longer pre-LAI antipsychotic duration and with a longer duration following the onset of psychosis.

At present, there are no specific guidelines regarding the early use of LAI antipsychotics [35], and this may contribute to their less frequent or delayed commencement [36]. There is evidence that suggests that olanzapine causes an increased appetite and weight gain [37], especially in patients with a lack of exercise in their routine [38], and a reduction in insulin sensitivity, leading to impaired glucose tolerance [39]. In our study, patients on olanzapine had significantly more frequent hyperglycemia and more frequent low HDL levels; hence, olanzapine should be used with caution in diabetic and obese patients.

The available literature has shown overall good patient outcomes and good patient compliance [40,41,42] and improvements in the quality of life of patients [43] with the use of the aripiprazole LAI. Based on our data, we found that patients on aripiprazole had a lower frequency of hyperglycemia or low HDL levels as well as hypokinesia. However, patients on aripiprazole presented significantly more frequent diastolic dysfunction compared to those on olanzapine or paliperidone.

As with any work, our study is not devoid of limitations. Firstly, it may not reflect the findings in a larger population with psychosis treated with LAI antipsychotics. Secondly, in our study, we could only focus on antipsychotics having LAI formulations to establish their correlations with cardiovascular risk factors. However, the overall approach towards patients with schizophrenia from their diagnosis, the initiation of medication, the use of oral medications, psychological tools, family support systems, and their exercise and dietary patterns have to be addressed in order to have a better understanding of the high cardiovascular risk burden in these patients. Moreover, further studies may be conducted with larger data sets. Not all schizophrenia patients receive monotherapy with LAIs. In addition, the drugs used for LAIs often change over the course of the evolution of a patient’s disease. The use of multiple LAI antipsychotics and their collective impact on patients’ cardiovascular status also have to be considered in future studies. Similar studies can be conducted in the future with a broader, multi-centric population, possibly with the inclusion of more groups, such as non-schizophrenic individuals, schizophrenic individuals not placed on LAIs but given OAs instead, and schizophrenic individuals not given any form of antipsychotic medication, for a comparison of their cardiovascular health outcomes. The literature has also shown subclinical chronic systemic inflammation and immune dysfunction as mechanisms behind the high cardiovascular risk in schizophrenia [44]. This calls for a study of such inflammation-/immunity-related factors and their association with cardiovascular comorbidities in schizophrenia patients.

## 5. Conclusions

We found strong links between several cardiovascular risk factors and the duration of psychosis; the duration of the LAI antipsychotic treatment; the duration between the onset of the disease and the start of LAI antipsychotic treatment; and the use of specific LAI antipsychotic medications. Abdominal obesity was more frequent among patients with a longer duration of psychosis (DP). Hyperglycemia was more frequent among patients with a longer duration before the onset of LAI antipsychotic use. Patients on olanzapine and risperidone had significantly more frequent hyperglycemia compared to those on aripiprazole or paliperidone. Hypertension was more frequent among patients with a longer duration between the onset of the disease and the start of the LAI antipsychotic treatment. The occurrence of hypokinetic heart disorders correlated positively with the duration of psychosis, the duration of the LAI treatment, and the duration between the onset of the disease and the start of LAI treatment. Patients on risperidone presented more frequent hypokinetic heart disorders. Echography findings of valvular changes as well as of diastolic dysfunction were more frequent among patients with a longer duration of psychosis and longer pre-LAI duration. Our study highlights the need for a better and more personalized cardiovascular care approach in patients with schizophrenia and also the need to explore the choice and timing of the LAI antipsychotic treatment that carries the lowest cardiovascular risk. Further research is needed before reaching any conclusion regarding the recommendation of any particular LAI antipsychotic with the “best” cardiovascular safety profile.

## Figures and Tables

**Table 1:** Clinical data regarding the duration of psychosis depending on the administered medication (LAI: long-acting injectable).

Antipsychotic		Age (upon Study Entry)	Onset Age	Months Passed since Onset: Duration of Psychosis (DP)	Time (Months) Passed in LAI Antipsychotic Treatment	Time (Months) Passed from Onset to LAI Antipsychotic Treatment Initiation
Olanzapine LAI	Mean	39.66	28.84	128.51	14.87	113.64
	N	39	39	39	39	39
	Std. Deviation	10.35	9.40	115.22	15.90	113.48
Risperidone LAI	Mean	43.77	29.74	169.67	26.29	143.38
	N	31	31	31	31	31
	Std. Deviation	11.08	11.53	138.40	20.92	131.86
Aripiprazole LAI	Mean	43.90	33.30	141.66	12.43	129.23
	N	30	30	30	30	30
	Std. Deviation	11.55	10.63	152.81	11.89	149.04
Paliperidone LAI	Mean	40.23	27.56	153.83	7.16	146.66
	N	30	30	30	30	30
	Std. Deviation	13.09	7.877	130.72	4.56	131.43
Total	Mean	41.75	29.79	147.20	15.25	131.95
	N	130	130	130	130	130
	Std. Deviation	11.51	10.03	133.04	16.09	129.99

**Table 2:** Number of patients in each dosage category for each antipsychotic drug.

		N	Percent %	Cumulative Percent
Valid	risperidone 75 mg/month	14	10.8	10.8
	risperidone 100 mg/month	18	13.8	24.6
	olanzapine 210 mg/month	2	1.5	26.2
	olanzapine 300 mg/month	8	6.2	32.3
	olanzapine 600 mg/month	20	15.4	47.7
	olanzapine 405 mg/month	8	6.2	53.8
	aripiprazole 400 mg/month	30	23.1	76.9
	paliperidone 150 mg/month	24	18.5	95.4
	paliperidone 100 mg/month	6	4.6	100.0
	Total	130	100.0	

**Table 3:** Number of months passed since onset of psychosis, months since LAI treatment initiation, and gap between onset of psychosis and initiation of LAI among different age groups.

Report				
Age Group		Months Passed since Onset: Duration of Psychosis (DP)	Time (Months) Passed in LAI Treatment	Time (Months) Passed from Onset to LAI Treatment Initiation
<30 years	Mean	37.40	9.25	28.15
	N	20	20	20
	Std. Deviation	27.89	7.80	28.21
Between 30 and 40 years	Mean	93.08	15.97	77.10
	N	37	37	37
	Std. Deviation	75.94	15.77	70.90
Between 40 and 50 years	Mean	148.94	16.69	132.25
	N	39	39	39
	Std. Deviation	109.46	18.49	106.38
>50 years	Mean	268.70	16.35	252.35
	N	34	34	34
	Std. Deviation	151.17	16.93	149.15
Total	Mean	147.20	15.25	131.95
	N	130	130	130
	Std. Deviation	133.04	16.09	129.99

**Table 4:** Number of patients in each age group that were given LAI antipsychotics, with most patients receiving olanzapine.

Crosstab						
Count						
		Antipsychotic				Total
		Olanzapine LAI	Risperidone LAI	Aripiprazole LAI	Paliperidone LAI	
Age groups	<30 years	5	3	4	8	20
	Between 30 and 40 years	16	7	6	8	37
	Between 40 and 50 years	11	14	10	4	39
	>50 years	7	7	10	10	34
Total		39	31	30	30	130

**Table 5:** Data regarding the cardiovascular risk factors.

	N	Minimum	Maximum	Mean	Std. Deviation
Body mass index	130	17.10	56.10	28.40	6.18
Abdominal circumference (cm)	130	63.00	150.00	96.16	16.26
Systolic blood pressure (mmHg)	130	90.00	189.00	126.55	14.63
Diastolic blood pressure (mmHg)	130	55.00	111.00	77.10	10.41
Glycemia (mg/dl)	130	65.00	271.00	113.06	30.51
Total cholesterol	130	76.00	387.00	167.48	49.82
Triglycerides	130	36.00	396.00	157.63	59.94
LDL-cholesterol	130	22.80	316.00	100.41	46.16
HDL-cholesterol	130	20.00	60.00	37.51	8.30
Number of cigarettes smoked daily	130	0.00	60.00	8.37	11.80

**Table 6:** Cardiovascular risk factors depending on the medication administered.

Antipsychotic		BMI	Abdominal Circumference (cm)	Systolic Blood Pressure (mmHg)	Diastolic Blood Pressure (mmHg)	Glycemia (mg/dL)	Total Cholesterol	Triglycerides	LDL-Cholesterol	HDL-Cholesterol	Number of Cigarettessmoked Daily
Olanzapine LAI	Mean	27.72	92.82	124.97	73.89	109.89	166.58	155.61	103.80	35.53	7.94
	N	39	39	39	39	39	39	39	39	39	39
	Std. Deviation	5.27	13.30	13.97	10.02	19.49	51.22	68.28	51.72	6.12	11.10
Risperidone LAI	Mean	29.23	99.87	131.45	78.22	120.67	149.22	170.29	85.87	34.45	10.48
	N	31	31	31	31	31	31	31	31	31	31
	Std. Deviation	5.24	14.03	11.915	8.71	30.15	44.34	68.80	40.85	8.03	14.68
Aripiprazole LAI	Mean	28.40	95.36	125.73	79.10	110.13	177.06	153.20	95.66	42.26	4.43
	N	30	30	30	30	30	30	30	30	30	30
	Std. Deviation	8.26	19.28	17.46	11.39	30.65	40.11	52.65	26.25	10.25	8.12
Paliperidone LAI	Mean	28.43	97.50	124.36	78.10	112.26	177.93	151.60	115.80	38.50	10.70
	N	30	30	30	30	30	30	30	30	30	30
	Std. Deviation	5.96	18.35	14.49	11.06	40.98	58.26	44.38	55.20	6.77	11.95
Total	Mean	28.40	96.16	126.55	77.10	113.06	167.48	157.63	100.41	37.51	8.37
	N	130	130	130	130	130	130	130	130	130	130
	Std. Deviation	6.18	16.26	14.63	10.41	30.51	49.82	59.94	46.16	8.30	11.80

**Table 7:** Changes in kinetics depending on the medication administered.

		Kinesia of the Left Ventricle		Total
		Normal	Kinetic Abnormalities	
Antipsychotic	Olanzapine LAI	33	6	39
	Risperidone LAI	17	14	31
	Aripiprazole LAI	23	7	30
	Paliperidone LAI	24	6	30
Total		97	33	130

## Data Availability

Data will be made available on valid written requests to the corresponding authors.
